# Comparisons of abdominal wall reconstruction for ventral hernia repairs, open versus robotic

**DOI:** 10.1038/s41598-021-86093-6

**Published:** 2021-04-13

**Authors:** Barbara Nguyen, Bryan David, Teisha Shiozaki, Kensey Gosch, G. Brent Sorensen

**Affiliations:** 1 Department of General Surgery, University of Missouri-Kansas City, Kansas City, MO USA; 2 St. Luke’s Hospital on the Plaza, Kansas City, MO USA

**Keywords:** Intestinal diseases, Outcomes research

## Abstract

The surgical complexities of our current population have pushed the technological limits of healthcare, urging for minimally invasive approaches. For ventral hernias, in particular, robotic assisted laparoscopic repairs have been met with conflict. Cost and longer operative times are among the arguments against robotic surgery, although thorough evaluation of patient outcomes could potentially advocate for use of this tool. We attempted to approach this by retrospectively reviewing our own data. We reviewed charts between September 2016 and February 2017 of patients receiving complex hernia repairs, either a standard open repair (SOR) or robotic-assisted repair (RAR). Data collected included preoperative, perioperative, and postoperative care. Of the 43 patients reviewed, 16 were SOR, versus 27 RAR. Patients were comparable in age, gender, BMI, diabetes as a comorbidity; average hernia defect size was similar between the two groups. Although operative times were longer in the RAR group, estimated blood loss (EBL) was less. Hospital stay was also shorter in the RAR group, at 3.0 ± 1.9 days versus 9.6 ± 8.4 days for the OAR group. Of those requiring critical care management, only one patient had a robotic assisted repair, versus half of the patients who received an open repair. Of the patients who presented to the emergency department within 30 days of surgery, each group had four patients, and two from the OAR group required admission. Our data is consistent with other literature supporting shorter lengths of stays. Although the robotic approach did required a longer operative time, the resulting improved patient outcomes support this technique for complex ventral hernia repairs.

## Introduction

Life expectancy, obesity rates, medical comorbidities—increased proponents within our patient population has given rise to complex ventral hernias, requiring unique approaches to repair. Over the last few decades, the approach to surgical repair has involved two major areas of focus: type of approach—such as open or laparoscopic—and physiologic alterations to repair, i.e. component separation. By expanding the width of coverage by means of retrorectus repair and posterior component separation, followed by placement of sublay mesh, improved coverage can be achieved^1^. Laparoscopic ventral hernia repair has increased in frequency in the past few decades. However, with wide complex hernias, the limited range of motion with laparoscopic instruments has not provided clear success over open approaches, and does not offer component separation capable with open repairs.

The emergence of robotic assisted repairs has challenged the limitations of complex hernia repairs. Abdominal wall reconstruction is a complicated field, and the learning curve for the robot is steep. Investing the time to master both is not without serious consideration. Unlike laparoscopic repairs, both open and robotic assisted laparoscopic repairs enable the surgeon to close the fascial defect. The ability to separate tissue planes with the robot allows for fascial closure with sublay mesh placement^1–5^. Robotic assisted laparoscopic surgery arguably provides the visualization and mobilization of tissues for component separation of open repair with the benefits of minimally invasive approach, with comparable outcomes. Recent literature of utilizing this approach at other institutions have demonstrated shorter length of stays, although longer operative times^2,6–8^.

Our institution has incorporated robotic assisted laparoscopic abdominal wall reconstruction over the last 2 years, while also continuing to perform open repairs. Our goal was to compare outcomes between the robotic and open approaches, with the primary objective as operative and hospitalization course differences. Secondary objectives included 30-day complications and readmissions. By exploring the differences in both patient and operative outcomes, we hoped to further elaborate and confirm the benefits of robotic surgery in complex ventral hernias.

## Methods

The preoperative selection process included multiple variables. Smoking cessation was required of all patients prior to receiving complex hernia repair. Pertinent demographics were reviewed included age, gender, diabetes, and body mass index (BMI).

Chart reviews were performed of patients undergoing complex abdominal wall repair from September 2016 to February 2017 at a single institution (St. Luke’s Hospital on the Plaza). We discussed with the Saint-Lukes IRB board if IRB approval was needed prior to proceeding with the study, but because the study was a retrospective review patient consent was not needed to be obtained. All methods were carried out in accordance with relevant guidelines and regulations. All repairs were performed by the same surgeon, with varying assistance, including general surgery residents and minimally invasive fellows. All of the patients undergoing the robotic assisted repairs underwent transverse abdominis plane (TAP) block pre-operatively. For the standard open repair (SOR), initial dissection was performed by the Rives–Stoppa–Wantz recto-rectus approach, followed by posterior component separation by means of transversus abdominis release (TAR). Placement of a sublay mesh was done after closure of the posterior layer. Two drains were routinely placed in the open repairs, one above the mesh and another in the subcutaneous space. Intraoperative data included defect size, operative time, estimated blood loss (EBL), and size and type of mesh used. Removal of either prior to discharge was dependent on output (< 30 mL). Pertinent components in chart review of hospitalization involved length of stay (LOS), any time requiring the intensive care unit (ICU) and critical care management, and complications prolonging hospital stay.

Due to the orientation of component separation with intra-abdominal dissection, all of the robotic assisted repairs (RAR) were TARs with posterior component separation. The technique, similar described in other literature, involves placement of three trocars on one side of the abdomen—usually the left first—with both rectus myofascial release and TAR dissection along the opposite side of the abdomen (right)^1,8^. After measurements, the appropriately sized mesh was placed within the abdomen, above the posterior layer, and anchored to the abdominal wall of the dissection side (right). The robot was docked to three new ports at the opposite side (right) and similar dissection performed (left side component separation). The hernia defect was then primarily closed. The posterior layer was primarily closed with the instruments still above this layer; thus, extraperitoneal. This enabled final suturing of the mesh to the opposite abdominal wall (left). No drains were placed.

At our facility, patient controlled analgesia (PCA) and an epidural are provided to the patient by anesthesia as standard acute postoperative care for the SOR group. While the anesthesia pain service is routinely involved with our SOR patients, we do not utilize this service or the associated pain control regimen (epidural and PCA) with the ROR group.

In addition to immediate postoperative complications, any complications within the first 30 days after surgery were reviewed, including those requiring re-admission or additional treatment. These were documented by emergency department visits, or evaluation in clinic, as all patients were seen 2 weeks from discharge. Although some complications did not require further intervention, such as seromas, they were documented in a separate category.

Descriptive statistics were summarized as means and standard deviations (SD) or medians and interquartile ranges (IQR) for continuous variables, and counts and percentages for categorical variables. Demographics, intraoperative, and hospitalization characteristics were compared between groups using the Students t-test or Wilcoxon Signed Rank test for continuous variables, as appropriate, and Pearson’s χ^2^ test for categorical variables.

### Ethical standards

IRB approval and patient consent was not needed to be obtained. All methods were carried out in accordance with relevant guidelines and regulations. Local Ethics committee was not involved.

## Results

Over the 17-month period, 43 patients were included in the study, with 27 RAR and 16 OAR. The patient population was predominantly Caucasian, with only three African–Americans, all in the RAR group. BMI average was 32.2 ± 6.4 in the SOR group, and 33.3 ± 5.5 for RAR. There were three diabetics in the OAR group, and seven in RAR. There were no statistically significant differences between the two groups for any of the demographics. The demographics and cumulative results of the two groups are shown in Table 1.

**Table 1:** Demographics and complications comparison.

	SOR n = 16	RAR n = 27	*P* Value
Age	55.4 ± 12.4	58.6 ± 10.4	0.367
**Sex**			
Male	4 (25%)	13 (48%)	0.133
Female	12 (75%)	14 (52%)	
**Race**			
Caucasian	16 (100%)	24 (89%)	0.282
African American	0 (0%)	3 (11%)	
BMI	32.2 ± 6.4	33.3 ± 5.5	0.558
Diabetes	3 (19%)	7 (26%)	0.719
Operative time	206.5	272.1	0.001
Estimated blood loss	146.9 ± 75.8	43.0 ± 85.1	< 0.001
Recurrence	9	15	0.964
**Mesh tube**			
Bard soft/ventralight ST/prolene soft	14	27	0.132
Strattice/phasix	2	0	
ICU	8	1	< 0.001
DVT	2	0	0.132
UTI	1	1	1.0
Clostridium difficile infection	1	0	0.372
SSI	2	1	0.544
OR	2	0	0.132
Abscess	1	1	1.0
Pneumonia	2	1	0.544
ED visit	4	4	0.443
Readmission	2	0	0.132

*SOR* standard open repair, *RAR* robotic assisted repair, *BMI* body mass index, *DVT* deep vein thrombosis, *UTI* urinary tract infection, *SSI* surgical site infection.

The average defect size was 242 square cm for the SOR patient population versus 216 square cm for the RAR group (p = 0.404, SD 189.8 ± 111.5). Synthetic mesh was used for all RAR patients and the majority of OAR patients. Patients with a history of wound or mesh infections received a biologic or biologic-derived prosthetic meshes, both of which were OAR patients.

In terms of perioperative comparisons, there was a statistically significant difference in average operative time, with the RAR group taking longer at 272.1 min, compared to an average time of 206.5 min with the OAR group (p < 0.001). Also statistically significant was the difference in estimated blood loss. While the open repairs average 146.9 ± 75.8 ml of blood loss, the robotic repairs had appreciably less, at 43.0 ± 85.1 ml (p ≤ 0.001).

Postoperatively, the length of stay differed at 9.6 hospital days for OAR versus 3.0 days for RAR (p ≤ 0.001). Of the 16 patients in OAR, eight required a duration of their hospital course in the critical care unit, compared to one in the RAR group (p ≤ 0.001). Reasons for admission to the ICU included hypotension and requiring mechanical ventilation. Three patients in the open group, required mechanical ventilation postoperatively. Both had the same number of patients—four—present to the emergency department within 30 days, although the only two that required readmission were in the OAR group (12.5%). One was admitted for shortness of breath and treatment of pneumonia, later diagnosed with *Clostridium difficile* colitis; the other was admitted for syncope, likely due to hypoglycemia.

## Discussion

With the increasing use of minimally invasive techniques, more studies have researched the outcomes of hernia repairs with different approaches. Our data looked at perioperative results, as well as postoperative outcomes. With similar demographics, including age, defect size, and body mass index, there were significant differences in multiple areas.

From a perioperative standpoint, our data showed statistically significant difference in regards to estimated blood loss. On average, the RAR group had 43 mL of blood loss vs 146.9 mL in the OAR group. The advantage of better visualization of the tissue planes theoretically enables quicker identification of bleeding, both pulsatile and oozing. Immediate and direct cauterization results in less time spent identifying the source, and thus less bleeding. Interestingly, perhaps the longer operative time may also correlate with more attention to detail of bleeding, although more likely it is related to adjustment of using the robot.

Although the RAR group had much less estimated blood loss, the operative times were longer. These results are similar to other studies^1,^^3,^^6,^^9^. The average operating time was 206.5 min for the OAR group, and 272.1 min for the RAR group. An important factor to consider is the novelty of robotic repairs in this group by the operating surgeon. While open abdominal wall reconstruction has been performed for several years, those receiving a robotic assisted repair were the first to be done at this facility. Our surgeon began incorporating robotic assisted approaches into practice shortly prior to proceeding with robotic wall reconstruction. The learning curve towards efficiency with robotic surgery remains variable, but was certainly quicker than open abdominal wall reconstruction. As the frequency of robotic repair increases, it is highly possible the operative time will decrease.

While the OAR group routinely received drains, the RAR group did not. Bitner, et.al had a similar study comparing open to robotic assisted repairs with similar results; however, a drain was placed for the majority of both patients, as well as the use of fibrin sealant^6^. Although we did have patients with seromas in follow-up—unsurprisingly more with patients who received a robotic assisted repair—these did not require further intervention and were not included as a complication. There is no definitive recommendation on the usage of drains, but could be a point of investigation in future studies.

Hospital length of stay was considerable lower in the RAR group. Our institution OAR length of stay average was 9.6 days versus the RAR group of 3.0 days (p < 0.001). In comparison to other literature for abdominal wall reconstruction, overall the number of days was increased in both groups. Other institutions have also noted decreased hospital stay length, although approximately 1–2 days for robotic repairs and up to 5 days for open repairs^2,6,8,10^. At our institution, those receiving open approaches are routinely given an epidural and pump-controlled analgesia (PCA), which may contribute to a longer hospital stay due to pain control weaning parameters and pain management expectations. Need for physical therapy and judicious advancement of diet were also factors considered in the open repair patients. Physical therapy was not required for the RAR patients, and a liquid diet was usually started soon after surgery, with advancement as tolerated. While some patients were able to be discharged by postoperative day one, two of the RAR patients developed an ileus, which prolonged their hospital stay for about a week.

Despite having longer operating times, there was a statistically significant number of patients in the OAR group compared to RAR who required critical care management. In comparison of our study sample, one patient in the RAR group required critical care versus eight patients in the OAR group (p < 0.001). The majority of these patients were monitored for either respiratory concerns or hypotension. The one RAR patient had been transferred for hypotension, and was discharged by postoperative day four.

Postoperative factors such as emergency department visits within 30 days with readmissions were also examined to further elaborate on potential differences in the subacute setting. Three of the four patients in the RAR group presented for seromas, none of which required intervention; the fourth presented with constipation. The OAR group also had four patients present to the ED within 30 days postoperatively. Two developed seromas that did not require intervention. Two developed postoperative small bowel obstruction that were managed nonoperatively.

Of the OAR group, 2 patients required surgical wound debridement for incision site breakdown while none of the RAR required re-operation and wound vac therapy. Other postoperative complications were reviewed such as occurrence of deep vein thrombosis, urinary tract infection, Clostridium difficile infection, surgical site infection, development of pneumonia without evidence of a statistically significant difference.

Limitations of this study include its retrospective design. This was a single site, single surgeon database; although open repairs at this institution have been ongoing for several years, the data included for robotic repairs are amongst the first performed by this surgeon, and at this institution. Despite this, similar results were still noted compared to other literature. Patient selection for OAR vs. RAR were based on surgeon and patient preference. There are no agreed guidelines for deciding between OAR vs. RAR asides from surgeon preference. Another possible bias arises that the larger defects, tended to be repaired in open fashion. Conclusions made in regards to these population are made with a short interval (within 1 year). Further studies need to be down for long-term follow-up and evaluation of possible hernia recurrence. Also, this study is based on the surgeon transitioning towards the robotic assisted technique. The study would benefit from a subsequent analysis after the surgeon’s experience and familiarity with robotic assisted laparoscopic cases increases. As this study is short term, another limitation is the accountability on hernia recurrence. Further studies will need to be completed to assess for recurrence rates on follow up.

Cost analysis would also be a crucial element to investigate in future studies. Belyansky suggested the additional operating time was more than offset by the reduction in hospital days, with overall costs for minimally invasive group being almost half of the open repair^2^. While operating times contribute heavily to costs, whether this outweighs the difference in hospital days could argue for one approach over the other. Accounting for overhead costs, particularly the expense of purchasing a robot in the early stages of use, is another factor to consider. The hospital costs for operating room needs and hospital rooms are financial concerns that could limit or favor the purchase of a robot.

## Conclusion

Ventral hernias have grown to be a significant source of morbidity in the population today and advances in the surgical field have allowed for newer techniques to be developed. Robotic assisted techniques are being more adopted into everyday practice. Our study shows that robotic assisted ventral hernia repair to be a viable option associated with lower total hospital days as well as lower perioperative complications. While length of operative time appears to be the most prohibitive factor in robotic repairs, further studies would be helpful in delineating if this improves with experience, as well as if overall costs favor one over the other. Decreased hospital lengths of stay and readmissions certainly encourage decreased costs, and patient outcomes within the first 30 days appear to globally better. It will be vital to continue investigation of long term outcomes, particularly of recurrence. Improved quality outcomes will be the determining factor for the permanence of robotic surgery; our study reinforces its role not only as a viable option, but as a mainstay in hernia repairs.
